# Surgery and Catheter Ablation for Atrial Fibrillation: History, Current Practice, and Future Directions

**DOI:** 10.3390/jcm11010210

**Published:** 2021-12-31

**Authors:** Patrick M. McCarthy, James L. Cox, Olga N. Kislitsina, Jane Kruse, Andrei Churyla, S. Chris Malaisrie, Christopher K. Mehta

**Affiliations:** 1 Division of Cardiac Surgery, Department of Surgery, Feinberg School of Medicine, Northwestern University, Chicago, IL 60611, USA; James.Cox@nm.org (J.L.C.); Olga.Kislitsina@nm.org (O.N.K.); jkruse@nm.org (J.K.); Andrei.Churyla@nm.org (A.C.); Chris.Malaisrie@nm.org (S.C.M.); christopher.mehta@nm.org (C.K.M.); 2 Bluhm Cardiovascular Institute, Feinberg School of Medicine, Northwestern University, Chicago, IL 60611, USA; 3 Division of Cardiology, Department of Medicine, Feinberg School of Medicine, Northwestern University, Chicago, IL 60611, USA

**Keywords:** atrial fibrillation, electrophysiologic mapping, catheter ablation, maze procedure

## Abstract

Atrial fibrillation (AF) is the most common of all cardiac arrhythmias, affecting roughly 1% of the general population in the Western world. The incidence of AF is predicted to double by 2050. Most patients with AF are treated with oral medications and only approximately 4% of AF patients are treated with interventional techniques, including catheter ablation and surgical ablation. The increasing prevalence and the morbidity/mortality associated with AF warrants a more aggressive approach to its treatment. It is the purpose of this invited editorial to describe the past, present, and anticipated future directions of the interventional therapy of AF, and to crystallize the problems that remain.

## 1. Introduction

**Figure 1:**
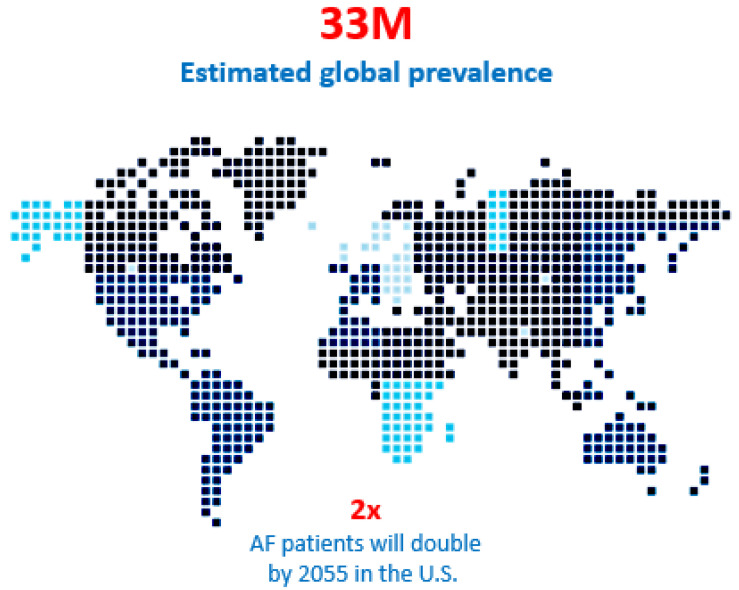
Atrial fibrillation is an epidemic and the patient population with an estimated 33 million people suffering from the anomaly globally and that number is predicted to double in the next 15 years. Moreover, 30% of those patients could benefit from interventional therapy, but only 9% are treated with catheter ablation or surgery annually.

**Figure 2:**
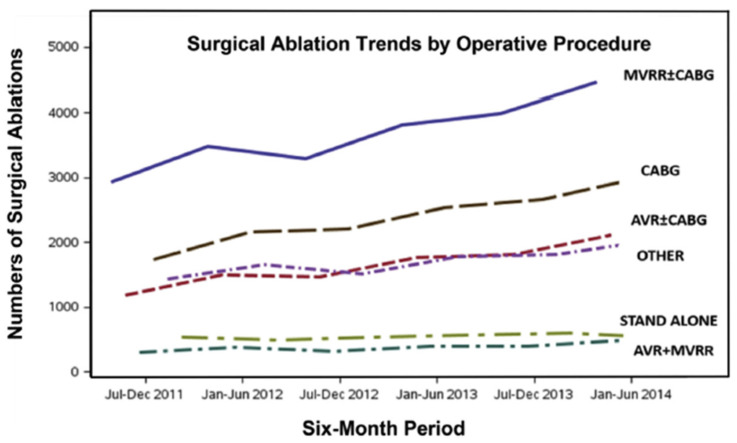
Trends in total numbers of surgical ablation procedures in the United States from 1 July 2011 through 30 June 2014. AVR = aortic valve replacement; CABG = coronary artery bypass graft surgery; MVRR = mitral valve repair or replacement (reproduced with permission from Badhwar et al.) [5].

Atrial fibrillation (AF) is a global epidemic affecting an estimated 33 million patients, a number that is expected to double over the next two to three decades (Figure 1) [1,2,3,4]. Only 3% of all AF is associated with other cardiac conditions (“concomitant” AF) that requires cardiac surgery such as coronary artery bypass grafting (CABG), mitral valve repair or replacement (MVR/r), and aortic valve replacement (AVR). In these patients, much less than half of them actually receive concomitant AF ablation. The vast majority of the remaining 97% of AF patients (“stand-alone AF”) are treated with drugs. While it is estimated that approximately 30% of those patients could benefit from interventional therapy, only 9% of them are currently treated by catheter or surgical ablation combined. The impact of surgery for AF is negligible in terms of its overall impact on this AF epidemic (Figure 2) [5] and catheter ablation is only marginally better. Although surgery is associated with a higher morbidity than catheter ablation, the latter is not without its risks (Table 1) [6].

**Figure 3:**
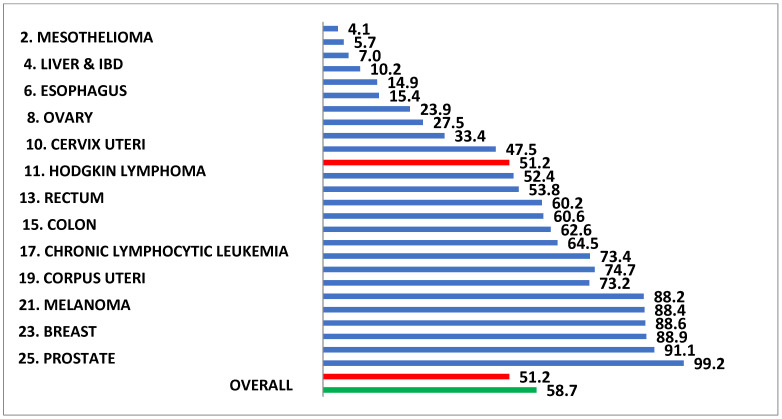
The life expectancy following the first in-hospital diagnosis of atrial fibrillation in Medicare patient ranks eleventh if compared to the life expectancy following the diagnosis of the 25 most lethal cancers in the United States. The life expectancy is shorter following the diagnosis of atrial fibrillation than it is for such common neoplasms as colon cancer, melanoma, and breast cancer.

**Figure 4:**
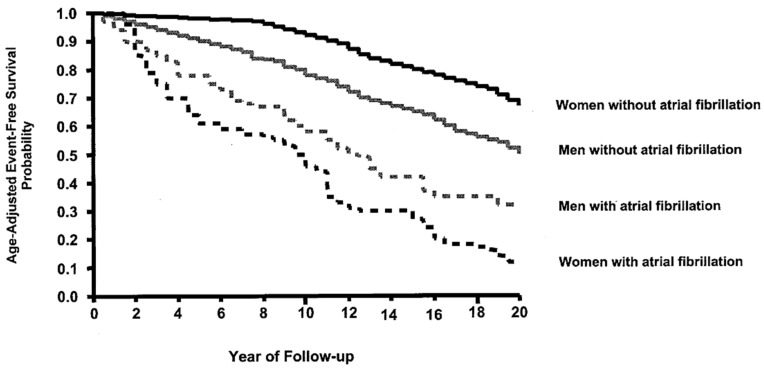
The impact of atrial fibrillation on the 20-year survival rate for women is much greater than the impact of atrial fibrillation in men (reproduced with permission from Stewart et al.) [10].

Perhaps because AF is the most common of all cardiac arrhythmias and occurs predominantly in the elderly, it is often considered less dangerous than other illnesses. However, the 5-year survival rate for AF would rank number 11 if ranked in a list of the 25 most deadly cancers in the US, well above colon cancer, melanoma, breast cancer, and 12 others (Figure 3) [7,8,9]. In addition, AF is much more lethal in women than in men (Figure 4) [10]. The increasing prevalence and the morbidity/mortality associated with AF warrants a more aggressive approach to its treatment, including the use of interventional therapy. It is the purpose of this invited editorial to describe the past, present, and anticipated future directions of the interventional therapy of AF, and to crystallize the problems that remain.

## 2. Surgical Intervention for Atrial Fibrillation

The first surgical “maze procedure” for the treatment of atrial fibrillation (AF) was performed on 25 September, 1987 [11]. It resulted in the restoration of normal sinus rhythm and the patient remained AF-free and on no anti-arrhythmia medications for 19 years, 11 months, and 1 week. Since that time, the maze procedure has undergone multiple iterations that have made it less invasive, quicker, and safer while retaining its status as the gold standard for the interventional treatment of AF.

Some 11 years after the first maze procedure was performed, less invasive radiofrequency (RF) catheter-based techniques were introduced [12] and they quickly became the preferred mode of interventional therapy. Over the past few decades, sophisticated new mapping techniques have further elucidated the underlying electrophysiology of AF and the optimal interventional therapeutic approach for individual patients has become clearer, though some points of controversy persist. 

### 2.1. Electrophysiologic Mapping of Atrial Fibrillation

**Figure 5:**
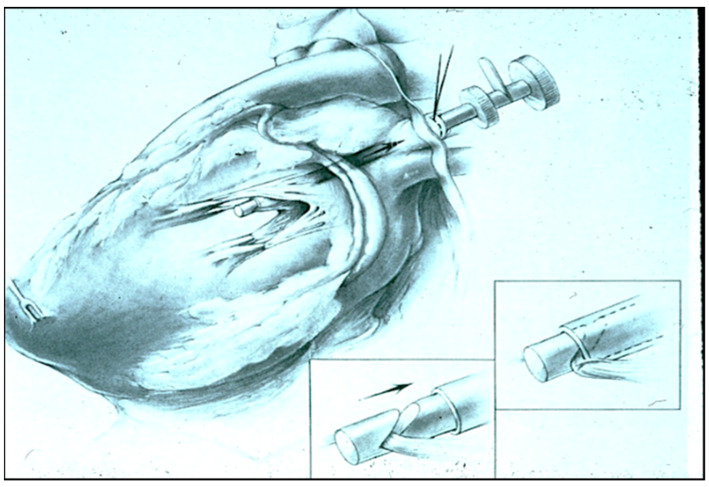
The experimental model created to map and treat atrial fibrillation in which mitral insufficiency is created without placing any lesions in the atrium and without causing any pericardial adhesions by violating the pericardial space.

The interventional ablation of AF was not feasible until it became possible to correlate three-dimensional atrial anatomy with three-dimensional electrical maps of atrial fibrillation in the mid-1980s. Prior to 1980, the electrophysiology of atrial fibrillation was studied primarily in isolated animal hearts using Langendorff perfusion models, hyperstimulation of the vagus nerves, or topical aconitine, an irritating solution that caused the atria to fibrillate. In an effort to develop a more clinically-relevant model of AF that could be used to evaluate potential ablative techniques, a canine model of AF secondary to chronic mitral valve insufficiency was devised in 1980 [13]. During the creation of experimental mitral insufficiency, great care was taken to avoid causing any type of atrial scarring or pericardial adhesions because they might artificially alter the electrophysiology of AF. Thus, under sterile operative conditions, a left thoracotomy was performed in dogs and a purse-string suture was placed in the left superior pulmonary vein (LSPV) in the left pleural space external to the pericardium (Figure 5). A high-fidelity Millar pressure catheter was inserted through this purse-string suture and passed into the left atrium (LA) for continuous monitoring of LA pressure. A Cope biopsy needle was then inserted through the same purse-string suture in the extra-pericardial LSPV and passed across the mitral valve into the left ventricle. The biopsy needle was then used to transect individual chordae tendineae of the mitral valve in a sequential manner until the LA pressure increased from its normal value of 2–3 mm Hg to approximately 20 mm Hg. The Millar catheter and biopsy needle were then removed, the purse-string suture was secured, and the left thoracotomy was closed. During the next several weeks, the LA enlarged significantly, though none of the animals developed spontaneous AF.

**Figure 6:**
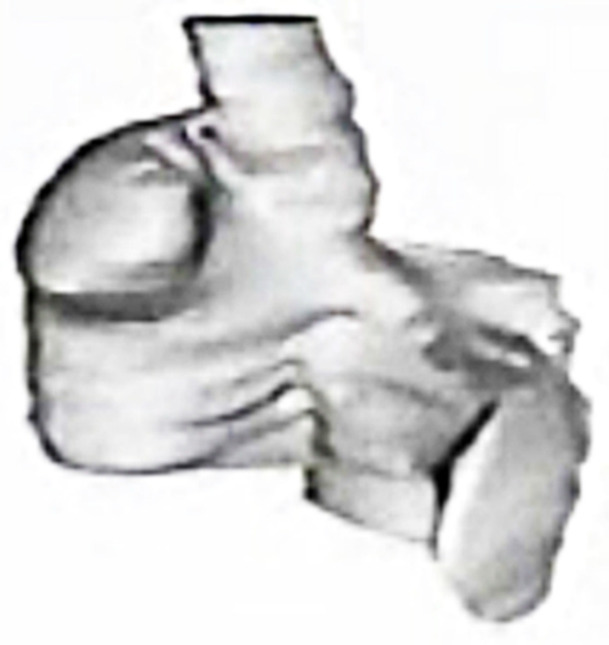
A three-dimensional anatomic reconstruction of the atria using gated ECG-MRI scans with each of the MRI slices “stacked” on one another. This figure is taken from a movie that was created in our experimental laboratories in 1986.

**Figure 7:**
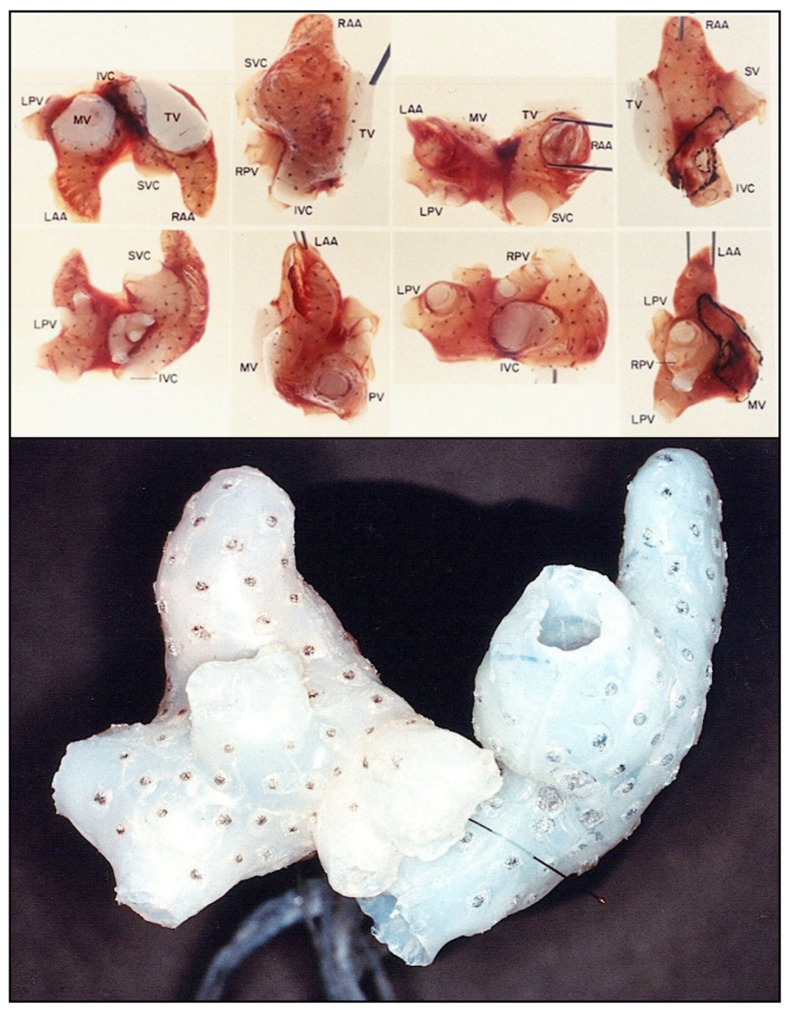
Three-dimensional electrode arrays used for the experimental mapping (upper panel) and clinical mapping (lower panel) of atrial fibrillation in the mid-1980s. The individual bipolar electrodes were newly designed “target electrodes” that had the anode as the central point and the cathode in a circular pole surrounding the central anode. This design eliminated potential artifact cause by differences in the direction of wavefront propagation. The three-dimensional electrical data was then superimposed on the three-dimensional anatomic reconstruction of the same atria using gated MRI scans (Figure 6). The experimental electrode arrays contained 256 individual bipolar electrodes and the clinical electrode arrays contained 156 individual bipolar electrodes. Both electrode arrays were designed and created by Dr. John P. Boineau and Dr. Richard B. Schuessler.

At times varying from 3 months to 3 years after the creation of mitral insufficiency, the animals were exquisitely sensitive to the electrical induction of AF, which often followed a single premature stimulus of the atria. In addition, while normal canine hearts can sustain AF for only a few seconds, if at all, the enlarged atria in our model typically sustained AF indefinitely until it was cardioverted, allowing prolonged electrical mapping of the AF to be performed. Gated magnetic resonance image (MRI) scans were used to create three-dimensional anatomic models of the atria (Figure 6) [14] and three-dimensional silastic electrode arrays containing as many as 256 individual bipolar electrodes (Figure 7) were then applied directly to the epicardial and endocardial surfaces of both atria to map the prolonged episodes of AF. Computer programs were developed in order to record the 256 electrograms simultaneously and continuously, providing the first three-dimensional electrophysiologic maps of atrial fibrillation. During this same period, human atrial fibrillation was mapped with 156 bipolar electrode arrays in the operating theater in patients undergoing surgery for the Wolff-Parkinson-White syndrome, 30% of whom also had episodes of clinical atrial fibrillation [15].

### 2.2. Development of the Maze Procedure for Atrial Fibrillation

The key information that these experimental and clinical studies provided was that atrial fibrillation was characterized by the presence of multiple large macro-reentrant circuits 5–6 cm in diameter in either or both atria [16]. If only one such circuit was present, the rhythm was atrial flutter. However, if more than one macro-reentrant circuit occurred simultaneously in either or both atria, the rhythm was atrial fibrillation. This observation that established episodes of both experimental AF and clinical AF were characterized by the presence of multiple, large macro-reentrant circuits presented the possibility that surgical interruption of those circuits might ablate atrial fibrillation.

It was clear from Moe’s experimental studies in the early 1960s [17] that dividing the atria into small segments could ablate experimental AF. The fact that the individual subsegments of atrium stopped fibrillating proved that AF was not sustained by focal mechanisms, otherwise the individual smaller segments would have continued to fibrillate. However, such compartmentalization of the atria into small isolated segments was useless clinically because it would preclude the ability of the isolated atrial segments not contiguous with the sinoatrial (SA) node to be activated. Thus, it was apparent that if surgically created lesions of conduction block (scars) were to be used to interrupt the macro-reentrant circuits responsible for AF, they specifically should not result in the compartmentalization of the atria into multiple isolated segments. Furthermore, it is essential that after AF ablation, both atria must be capable of being activated by an impulse generated in the SA node, dictating that the entire atrial myocardium would have to remain electrically contiguous. The only way to accomplish this result was to place the surgical lesions in a maze pattern with the lesions close enough to preclude the development of macro-reentrant circuits while leaving the atrial myocardium contiguous with the SA node and AV node to assure postoperative atrial function and electromechanical A-V synchrony of the heart.

**Figure 8:**
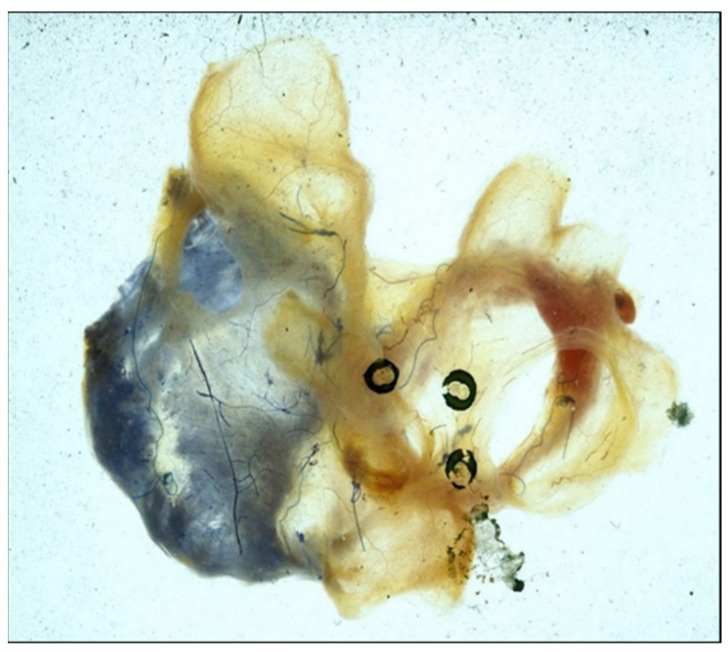
Photograph of both atria immediately following the performance of a maze procedure in a canine experiment. The right coronary artery was injected with blue dye and the left coronary artery was injected with red dye to detect any evidence of devascularization of either atrium. There was none.

**Figure 9:**
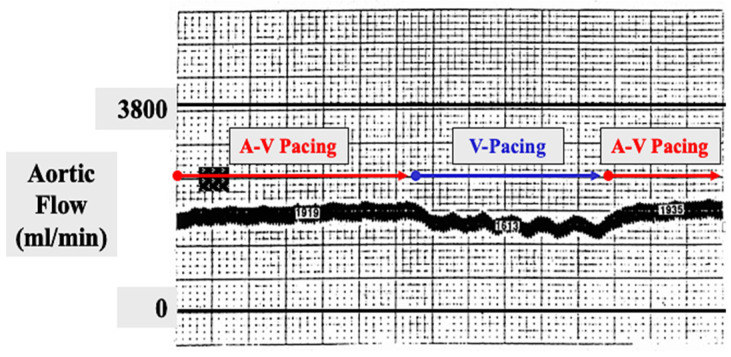
In order to document that atrial transport function persisted following an experimental canine maze procedure, an electromagnetic flow probe was placed around the ascending aorta. A-V sequential pacing was initiated and the aortic flow was recorded and remained stable for several minutes. The atrial pacing wire was then suddenly disconnected from the pacemaker, leaving the heart being paced in the ventricle only with no atrial contribution to forward aortic flow. The aortic flow immediately decreased by approximately 20% during ventricular pacing only. The atrial pacing wire was then reconnected to the pacemaker to resume A-V sequential pacing. The aortic flow immediately returned to its normal level, proving that the atrial contribution to forward aortic blood flow was approximately 20% following a maze procedure.

A maze pattern of atrial lesions was envisioned and immediately applied to the atria of the canine atrial fibrillation models described above. In terms of ablating the AF, this so-called “maze procedure” was successful in all experiments. However, due to the concern that the multiple atrial lesions of the procedure might devascularize the atria or otherwise injure their ability to contract postoperatively, exhaustive experimental evaluation was undertaken. Fortunately, those experiments documented that the atria were not devascularized by the maze procedure (Figure 8) and that the transport function of both atria remained intact postoperatively (Figure 9). The maze procedure was then applied clinically in the first patient mentioned above, and then in one patient every 6 months for the next 3 years until institutional IRB approval was granted to perform it whenever indicated clinically.

### 2.3. Chronology from the Maze-I to Maze-IV Procedures for Atrial Fibrillation

**Figure 10:**
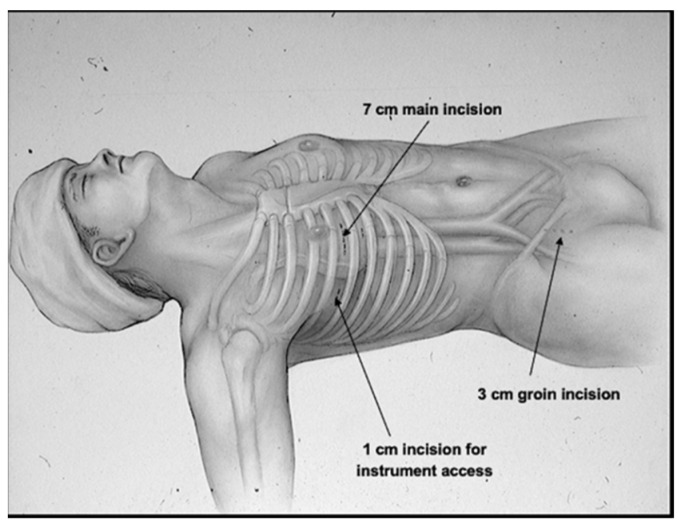
In 1997, the first minimally invasive surgical procedure for atrial fibrillation was performed. Because the far left side of the left atrium, which often had a bleeder from a branch of the circumflex coronary artery following a standard cut-and-sew maze procedure, could not be reached through the small mini-thoracotomy in the right fourth intercostal space, all of the lesions of the minimally invasive maze procedure had to be created with a cryoprobe rather than with a scalpel or scissors. This procedure was called the minimally invasive cryo-maze III procedure, and it has remained largely unchanged for the past 25 years (reproduced with permission from Cox JL) [18].

Following the initial publication of the details of the original maze procedure in 1991, it was performed on a routine basis in several leading medical centers worldwide but because of its complexity, it was not accepted as a feasible interventional procedure for the treatment of AF by most surgeons and cardiologists. Indeed, during the first 3–4 years of the 1990s, the only two surgeons in the world who performed the maze procedure on a routine basis were the senior author (J.L.C.) at Barnes Hospital/Washington University in St. Louis where it was developed and the first author (PMM) then at the Cleveland Clinic. Between September 1987 and April 1992, two separate iterations of the original maze pattern of lesions (maze-I) were utilized, eventually resulting in the final pattern of the maze-III procedure. In 1997, the original “cut-and-sew” maze-III procedure was converted to a minimally invasive cryosurgical maze-III procedure that was not only less invasive but also attained the same results with fewer complications than the original cut-and-sew maze-III procedure (Figure 10) [18].

**Figure 11:**
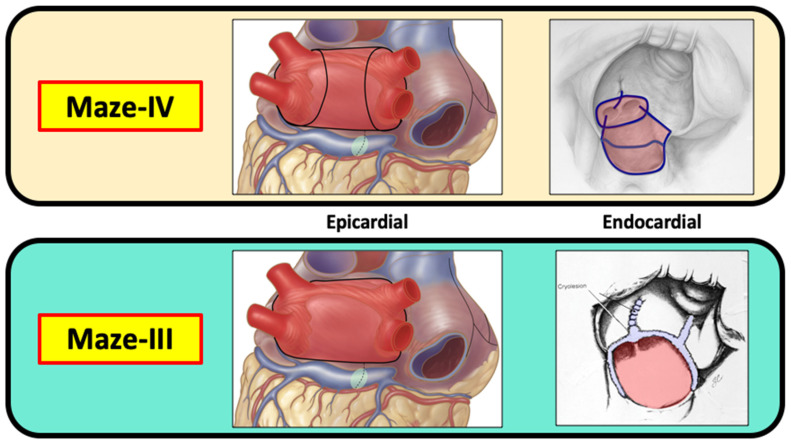
Both the maze-III and the maze-IV create a “box lesion” around all four pulmonary veins and include isolation of the posterior wall of the left atrium. Although the box lesion is created in a different manner in the maze-III and the maze-IV, the end result is exactly the same from an electrophysiological standpoint. All other lesions in the maze-III and the maze-IV are the same. Therefore, there is no difference between the maze-III and the maze-IV in terms of their effectiveness in treating atrial fibrillation.

Both the original cut-and-sew procedure, which was performed via a median sternotomy, and the totally cryosurgical procedure that was performed via a mini-thoracotomy in the right fourth intercostal space were maze-III procedures with identical lesion patterns. Because the lesion patterns remained unchanged, the minimally invasive cryosurgical procedure was not designated with another Roman numeral as the “maze-IV” procedure for fear of causing confusion. The current maze-IV procedure was introduced in 2002 by Damiano and associates [19], and differs from the maze-III only in the manner in which the pulmonary veins are encircled (Figure 11). The main advantage of the maze-IV procedure over the maze-III procedure is that in most surgeons’ hands, the maze-IV procedure can be performed with shorter aortic cross-clamp times.

## 3. Catheter Ablation for Atrial Fibrillation

The pre-maze procedure experimental and clinical mapping studies described above documented that once an episode of AF is generated, it is sustained by multiple, self-perpetuating atrial macro-reentrant circuits. This concept has often been interpreted as being the same as the older, debunked “multiple wavelet theory” of atrial fibrillation, though the macro-reentrant concept has nothing to do with wandering “multiple wavelets”. The clinical success of the surgical maze procedure over the past 30-plus years that is designed specifically to preclude or interrupt the multiple macro-reentrant circuits responsible for sustaining AF, confirms the validity of this electrophysiological concept of how atrial fibrillation is sustained. However, those initial studies failed to detect the presence of the atrial triggers that induced the episodes of AF.

### 3.1. Evolution of the Therapeutic Goals of Catheter Ablation for AF

In 1998, Haissaguerre et al. [12] reported that each individual episode of paroxysmal atrial fibrillation is induced by atrial “triggers” and that 90% of those triggers are located in the pulmonary veins. His study also showed that the frequency of occurrence of the paroxysmal episodes of AF could be drastically decreased or eliminated entirely by isolating these triggers from the rest of the atrium using a new interventional technique, radiofrequency (RF) catheter pulmonary vein isolation (PVI), which was vastly less invasive than the surgical maze procedure, which requires cardiopulmonary bypass even when performed by minimally invasive surgical techniques. Thus, Haissaguerre’s seminal article provided both a more complete understanding of the electrophysiology of AF and a much less invasive way to treat its paroxysmal form (PAF), which represents slightly over one-half of all patients suffering from AF. 

Unfortunately, many readers seemed to overlook the fact that nearly one-half of AF patients suffer from prolonged, non-intermittent AF, not from paroxysmal episodes of AF. However, there was no suggestion in the Haissaguerre article that PVI alone should be applied for any type of AF except the paroxysmal type (PAF) or that pulmonary vein triggers were responsible for sustaining AF in patients in its more prolonged, persistent forms. Nevertheless, following the Haissaguerre article, both interventional electrophysiologists and surgeons immediately began to perform catheter PVI for all types of AF, including long-standing persistent AF, and our industrial colleagues quickly geared up to create surgical devices that had only one purpose—to encircle the pulmonary veins. Not surprisingly, it soon became apparent that simple isolation of the orifices of the pulmonary veins (PV’s) was not 90% effective for the treatment of paroxysmal atrial fibrillation (PAF) as it theoretically should have been if 90% of the triggers for AF were located within the PV’s [20]. In addition, the inadequacy of PVI alone as an optimal treatment for the more prolonged, sustained types of AF quickly became clear. As a result, the target areas for catheter ablation in the left atrium were extended beyond the PV orifices themselves to include the “antrum” of left atrial myocardium surrounding the PV orifices [21]. This enlargement of the atrial target areas resulted in an improvement in the success rates for AF ablation, but an attempt to encompass even more of the posterior wall of the LA was largely abandoned because of an increase in esophageal injury that caused lethal atrioesophageal (AE) fistulas [22]. Interventional electrophysiologists eventually settled on performing PVI’s that included as much of the surrounding antral areas as possible without risking esophageal injury. Several measures were used to decrease the likelihood of esophageal injury during catheter ablation, including manually “displacing” the esophagus during the procedure with an indwelling esophageal probe [23]. Other approaches have included intra-luminal esophageal cooling devices [24].

### 3.2. The Importance, or Lack Thereof, of Real-Time AF Mapping

It is noteworthy that during this two-decade evolution in the specific therapeutic goals to be attained during the catheter ablation of AF, the one consistent objective cornerstone has been isolation of the pulmonary veins, a procedure that is based on anatomy, not on electrophysiology. During the same period, ingenious electrophysiologic mapping techniques have evolved that have improved our understanding of atrial fibrillation but in most cases, they have been used primarily to document the completeness of catheter ablation lesions in and around the pulmonary veins. More recently however, some clinical investigators have attempted to position the atrial ablative lesions on the basis of real-time atrial electrophysiologic maps and to ablate specific areas of the atrium exhibiting continuous fractionated electrical activity (“CFAE ablation”) [25], as well as small independent “rotors” of electrical activity believed to be responsible for sustaining AF in its more persistent forms [26]. 

The persistent question is whether interventional procedures to ablate atrial fibrillation can be “map-guided” as they are for less complex arrhythmias like the Wolff–Parkinson–White (WPW) syndrome, AV node reentry tachycardia, automatic atrial tachycardia, and many types of ischemic and non-ischemic ventricular arrhythmias. Thus far, the answer would seem to be “no”. Unlike those other simpler arrhythmias, the attempt to “tailor” interventional procedures to the treatment of specific electrophysiologic findings in different patients has proven to be frustrating at best and futile at worst. Thus, the only two interventional procedures that have proven to be consistently optimal for the treatment of AF over the past three decades are: (1) PVI for paroxysmal AF, and (2) the maze procedure for all types of AF, both of which are based on anatomic structures, not on electrophysiologic guidance, because neither requires that individual lesions be positioned on the basis of real-time electrophysiologic maps [27]. This is not meant to suggest that attempts at the map-guidance of AF should be abandoned but rather, to point out that despite our ability to map atrial fibrillation in the most elegant and detailed manner today, the impact of map-guidance on its specific interventional treatment remains negligible.

Perhaps the major difference in atrial fibrillation and all other cardiac arrhythmias is that its electrophysiologic patterns are unstable, often fleeting in nature, and can change in relation to underlying anatomy from one instant to the next. Indeed, recent unpublished data in which separate body-surface maps were recorded sequentially in the same AF patients 10 days apart showed dramatic differences in the electrophysiologic patterns of the AF in the same patient at different time intervals [28]. Since the ablation of specific atrial sites exhibiting electrophysiologic abnormalities (CFAE sites; small local rotors, etc.) change anatomic locations over time, their focal ablation at the time of mapping are likely to be ineffective because they can simply reappear later at some other anatomic site. Such is the devilish nature of persistent atrial fibrillation, making it problematic to “map-guide” its interventional treatment. This problem was first recognized in the mid-1980s and it was the principal reason that instead of trying to “map-guide” the surgical treatment of AF, as was routine at the time for all of the other less complex arrhythmias, a “fallback” approach was pursued in which all patients with AF received the same anatomy-based surgical intervention, the maze procedure.

### 3.3. Fundamental Problems with Catheter Ablation for AF

A misconception of catheter ablation that has consistently caused confusion is the discrepancy between the sites of intended ablation and the sites of actual ablation. This has often resulted in inaccurate conclusions being drawn regarding the relationship between atrial anatomy and AF electrophysiology. For example, it has been demonstrated repeatedly that failure to isolate the PV’s permanently does not necessarily mean that recurrent AF will ensue. For example, a high incidence of pulmonary vein “reconnection” has been documented within a year after a catheter PVI procedure [29]. This led many to conclude that complete, permanent isolation of the PV’s might not be essential to success for the treatment of PAF as if somehow, partial PV isolation was sufficient. However, an alternative explanation is that the PVI procedure is successful in such patients because it is not actually an isolation procedure but rather, an ablative procedure, one in which the RF lesions in the LA inadvertently ablate most of the atrial triggers near the PV’s rather than isolating them. There is nothing inherently wrong with that inadvertent approach but ablating the triggers rather than isolating them leads to misconceptions of how catheter ablation actually works to rid patients of PAF and those misconceptions can have undesired consequences (see STAR-AF II discussion below).

**Figure 12:**
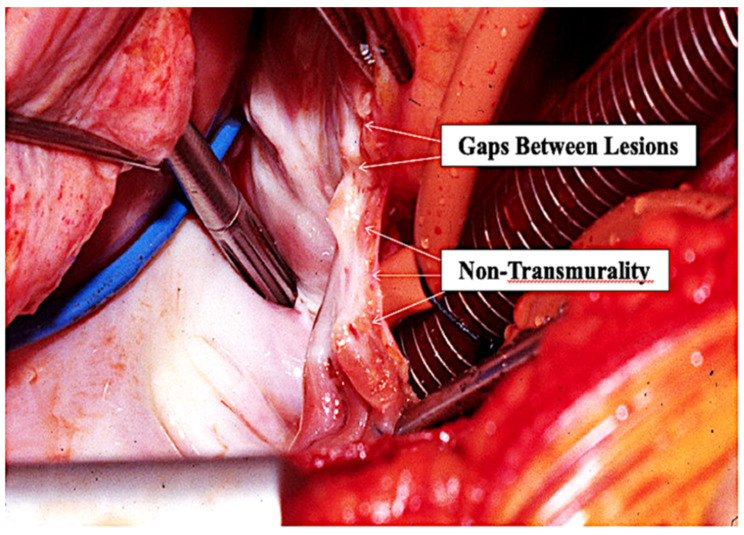
Operative photo of a patient who has had a previous endocardial catheter ablation for atrial fibrillation. The gaps between the individual lesions created by the tip of the RF catheter and the non-transmurality of most of the endocardial catheter lesions is obvious.

This concept of catheter PVI being as much an ablative procedure as an isolating procedure is supported by the location and distribution of the actual atrial endocardial RF scars that can easily be seen by surgeons in post-catheter ablation patients who require subsequent AF surgery. Surgeons commonly describe the inside of the LA in such patients as looking like it had been “carpet-bombed” because of the random location of the ablation scars throughout the posterior LA. It is undeniable that the atrial endocardial scars end up being located where the atrial endocardial RF energy was actually applied. These lesions are typically neither contiguous with one another nor uniformly transmural (Figure 12). Again, this discrepancy led to the conclusion by many that complete transmurality/contiguity of the ablation lesions is not essential to long-term conduction block, a notion that is completely inconsistent with proven anatomic–electrophysiologic principles. For example, we have known for over 50 years that a single accessory pathway (which is actually a strand of atrial muscle) responsible for the reciprocating tachycardia associated with the WPW syndrome need be only the size of a human hair in order to conduct electrical activity. Others have shown that only 1 mm of atrial myocardium will conduct electrical activity. Therefore, any “gap” remaining between individual catheter ablation lesions or non-transmural endocardial lesions that viable subepicardial atrial myocardium above a permanent endocardial lesion will most certainly result in electrical conduction across any intended “line” of endocardial catheter ablation lesions. In addition, to our knowledge, no surgeon has ever identified any combination of RF catheter lesions that form an actual “line” inside the LA following catheter ablation for AF. As Haissaguerre himself once said to one of the authors (JLC), “Trying to create a line in the atrium with the tip of a catheter is like trying to cut off the corner of a piece of paper using a straight-pin…it’s possible, but it is very difficult”. Both the Stereotaxis system [30] and the Hansen robotic system [31] were designed to make it easier to create individual atrial RF lesions in a contiguous line, but neither significantly improved the results of catheter ablation for AF. 

**Figure 13:**
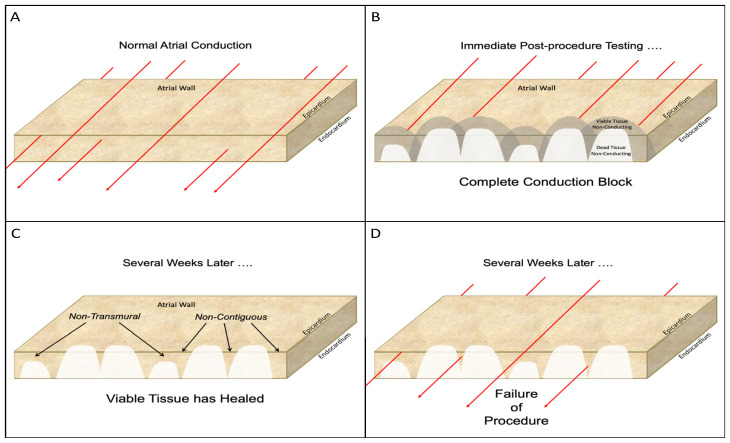
Schematic diagram of the problems associated with catheter ablation for atrial fibrillation. (**A**): Normal electrical conduction through the atrial wall. The red lines represent the electrical conduction in all diagrams. (**B**): Following the completion of the catheter ablation, immediate post-procedure testing shows complete conduction block due to the endocardial lesions. However, undetectable at this time, is the presence of both dead myocardium and damaged, but viable, adjacent tissue. Electrophysical testing at this time can be misleading because neither the dead tissue nor the adjacent damaged, but viable, tissue is capable of conducting electrical activity. (**C**)**:** in some cases, the viable tissue will recover with time and resume its ability to conduct electrical activity. This recovery of the damaged, but viable, tissue leaves lesions that are neither contiguous (i.e., lesions with “gaps”) nor uniformly transmural. (See Figure 12). (**D**): such non-transmural, non-contiguous lesions result in resumption of electrical conduction across the desire line of conduction block and failure of the catheter ablation procedure.

Even if a “line” of RF lesions can be created more accurately using these systems, neither of them addresses the problem of non-transmurality of endocardial RF lesions. The inability to be certain that a given endocardial RF catheter lesion is transmural has plagued interventional electrophysiologists since the advent of catheter ablation for AF. While intra-procedural electrical “testing” may show complete isolation of the PV at the time of the ablation procedure, it doesn’t necessarily mean that the lesions will all remain transmural once they have healed (Figure 13). The lesions may appear to be transmural shortly after they are created because electrical conduction cannot occur across irreversibly injured tissue or across viable tissue that has been temporarily rendered non-conductive because of temporary sublethal injury of some of the adjacent myocardial cells. Once subsequent healing allows the injured but viable myocardium to resume electrical conduction, the procedure fails. The problem of non-transmurality was addressed most aggressively by the introduction of “contact-force” catheter tips that can measure the force exerted on the endocardium by the tip of the ablating catheter [32]. This improved the percentage of transmural lesions following catheter ablation but it was initially accompanied by an increase in AE fistulas [33]. That problem was largely overcome by avoiding the creation of atrial lesions near the esophagus, but the inability to create contiguous, uniformly transmural linear lesions in the atria with a catheter remains one of the major stumbling blocks to optimal efficacy.

The problem of interpreting the results of catheter ablation caused by these deficiencies was perhaps most obvious in the STAR-AF II trial [34] in which the addition of catheter-created “lines” to pulmonary vein isolation (PVI) had no effect on the success of AF ablation. The study completely ignored the fact that, as Haissaguerre said, it is extremely difficult to create a contiguous, uniformly transmural line in the atrium with a catheter tip. In addition, one can be relatively certain that the inside of the left atrium in the three groups compared (PVI only, PVI with CFAE ablation, and PVI with additional “lines”) looked very similar once the lesions had healed, suggesting that all three groups in the study had essentially the same ablative procedure. Thus, the fatal flaw in the STAR-AF II trial was introduced with its initial design, which erroneously assumed that all of the atrial “lines” were uniformly contiguous and non-transmural. This erroneous assumption automatically negated the validity of comparing PVI with and without “lines”. The results of this famous and impactful trial led to the counter-intuitive, and utterly indefensible, conclusion that “less is more” in the catheter ablation of atrial fibrillation!

### 3.4. Results of Catheter Ablation for Atrial Fibrillation

There are few, if any, developments in clinical cardiology over the past 50 years that are more impressive than the success that can now be achieved by catheter ablation for virtually all cardiac arrhythmias except the persistent forms of atrial fibrillation. The fact that catheter ablation is extremely successful for the WPW syndrome (which once took surgeons several hours of open-heart surgery to accomplish), AV node reentry tachycardia, automatic atrial tachycardia, ischemic ventricular tachycardia (which once took surgeons all day to accomplish with very high-risk surgery), non-ischemic ventricular tachycardia, atrial flutter and paroxysmal atrial fibrillation cannot be denied. Moreover, it is also amazing that multiple catheter ablations are also successful (at 5-years) in nearly one-half of all patients with long-standing persistent AF [35]. That would be an unacceptable outcome for cardiac surgery, but for a procedure that is as minimally invasive, such as a catheter ablation, it is nothing short of remarkable. Nevertheless, there is still plenty of room for improvement, which will almost certainly come with the development of the appropriate tools for the catheter ablation of these more complex forms of atrial fibrillation. In the meantime, we are left with the current status of catheter ablation for AF.

When discussing the effect of any interventional procedure for AF, it is critical to separate the results into PAF and non-PAF (persistent and long-standing persistent AF) and it is helpful to further divide the latter into persistent AF and long-standing persistent AF when possible. However, most studies, including those reporting on catheter ablation and surgical ablation, have not done that. In addition, the results of AF catheter ablation and surgical studies have been reported too often as 1-year results. That is much like reporting the 1-year results of surgery for pancreatic cancer, which is quite good at 1 year but abysmal at 3 years. Additionally, outcome studies are published or ongoing to evaluate ablation strategies related to energy sources and lesion sets in targeted populations to further tailor patient treatment [36,37,38,39]. Finally, catheter ablation usually is not used in patients with severe left atrial enlargement.

**Figure 14:**
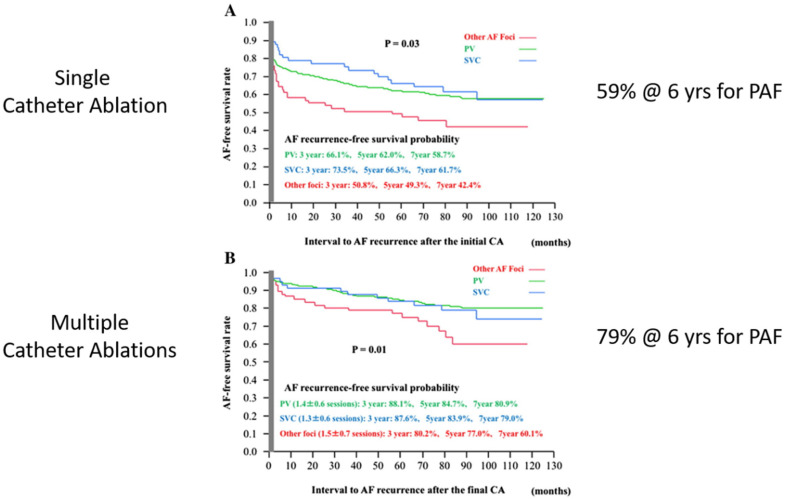
(**A**) A single catheter ablation for paroxysmal atrial fibrillation (PAF) is successful approximately 60% of the time. (**B**) Multiple catheter ablations for paroxysmal atrial fibrillation (PAF) are successful approximately 80% of the time. (Reproduced with permission from Takagawa) [40].

**Paroxysmal atrial fibrillation:** in 2017, Takagawa et al. reported a meta-analysis of the results of catheter ablation for PAF [40]. The results showed success rates for a single catheter ablation for PAF of 59% and for multiple catheter ablations of 79% at 6 years (Figure 14). Thus, the generally accepted success rate for the catheter ablation of PAF is approximately 80%.

**Persistent atrial fibrillation:** based on the known electrophysiology of atrial fibrillation, there is little reason to think that PVI alone would be optimal for the treatment of persistent or long-standing persistent AF. Yet, in 2017, a meta-analysis of 14 studies of 956 patients showed a 1-year success rate of 66.7% after performing only a PVI for persistent AF [41]. In 2018, Akkaya et al. reported a 1-year success rate for PVI using the second-generation cryoballoon of 76.2% and a 5-year success rate of 67.9% for persistent AF in 218 patients [42]. Thus, the generally accepted success rate for the catheter ablation of persistent AF is approximately 67%.

**Mixed populations of PAF and non-PAF:** in 2006, Calkins’ group reported the 2-year results of catheter ablation for a mixed group of patients (“all comers”), 48% of whom had PAF and 52% of whom had non-PAF [43]. The 2-year success rates were 28% for single catheter ablation and 68% for multiple catheter ablation. The first overall 5-year results for the catheter ablation of AF were reported by Haissaguerre’s group in 2011 [44]. They showed that in a series of “all comers” with AF (48% PAF, 52% non-PAF), the 5-year success rate following a single catheter ablation was 29% and following multiple catheter ablations it was 63%. Tondo and the Clinical Service 1STOP project investigators showed that in 486 patients from multiple centers, the success rates for a single PVI using cryoballoons in patients with persistent and long-standing persistent AF were 63.9% at 1-year and 51.5% at 18 months [45]. In 2019, a multi-center randomized clinical trial of contact-force RF ablation and two different regimens of cryoballoon ablation resulted in no difference in 1-year AF therapeutic efficacy [46]. Thus, the current generally accepted success rate for the catheter ablation of “all comers” with a diagnosis of atrial fibrillation is approximately 60%. Interestingly, this number has not changed in 20 years.

**Figure 15:**
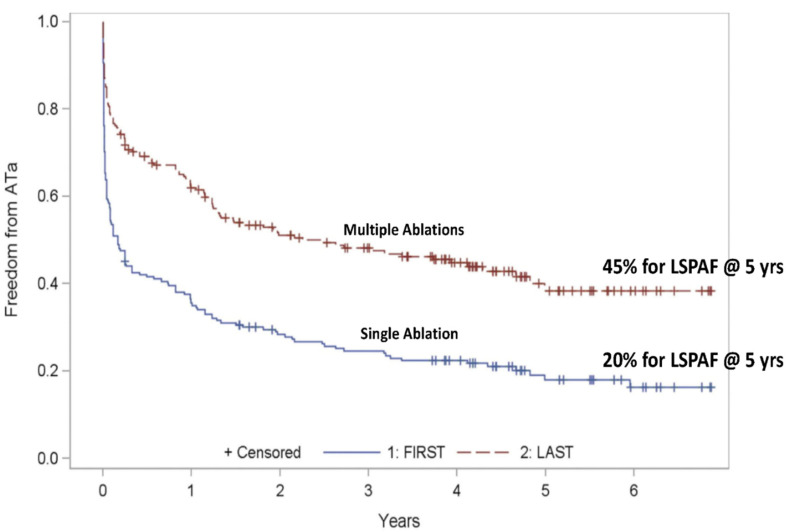
Single catheter ablation for long-standing persistent atrial fibrillation (LSPAF) has a 5-year success rate of 20%. Multiple catheter ablations for long-standing persistent atrial fibrillation (LSPAF) have a 5-year success rate of 45% (reproduced with permission from Tilz) [35].

**Long-standing persistent atrial fibrillation:** the major study of catheter ablation for long-standing persistent AF with a 5-year follow-up was from Kuch’s group, who reported the results of the Hamburg trial in 2012 (Figure 15) [35]. The 5-year success rates were 20% for single catheter ablation and 45% for multiple catheter ablations with a success rate of 40% for multiple catheter ablations at 6 years. Thus, the generally accepted success rate for the catheter ablation of long-standing persistent AF is approximately 40%.

Thus, despite the multiple advances in catheter technology and mapping capabilities introduced over the past 20 years, millions of treated patients, and billions of dollars spent on the problem, catheter ablation has proven to be the procedure of choice for PAF, but it still fails in roughly one-half of non-PAF patients, representing almost one-half of all patients with atrial fibrillation.

## 4. Current Status of Surgical Ablation for Concomitant AF

With few exceptions, cardiac surgeons deal almost exclusively with atrial fibrillation that is associated with other cardiac conditions that require surgery, including patients undergoing mitral replacement or repair (MVR/r), coronary artery bypass grafting (CABG), aortic valve replacement (AVR/r), or some combination of these procedures. This type of AF has been termed “concomitant AF” and is to be differentiated from so-called “stand-alone AF” in which AF occurs in the absence of associated cardiac abnormalities that warrant surgery. Stand-alone AF is treated interventionally primarily by catheter ablation, though rarely cardiac surgeons also treat stand-alone, while concomitant AF is treated solely by cardiac surgeons. In addition, most patients with concomitant AF have very dilated atria (especially with mitral disease) and based on left atrial size would not be considered candidates for catheter ablation.

In 2002, Damiano and colleagues described the maze-IV procedure in which the lesions of the maze procedure are performed with a combination of RF and cryosurgery [19] The lesion pattern of the maze-IV procedure differs from that of the maze-III procedure [47] only in how the pulmonary veins and “box lesion” are performed. However, the electrophysiological results of the maze-III and maze-IV lesion patterns are identical. The proposed advantages of the maze-IV procedure over the maze-III procedure are that it can be accomplished with a shorter aortic cross-clamp time and many surgeons find it to be easier to perform than the maze-III procedure. For those reasons, the maze-IV procedure is now the most commonly performed operation for concomitant AF.

### 4.1. Prevalence of AF in Cardiac Surgery Patients and Barriers to Its Treatment

**Figure 16:**
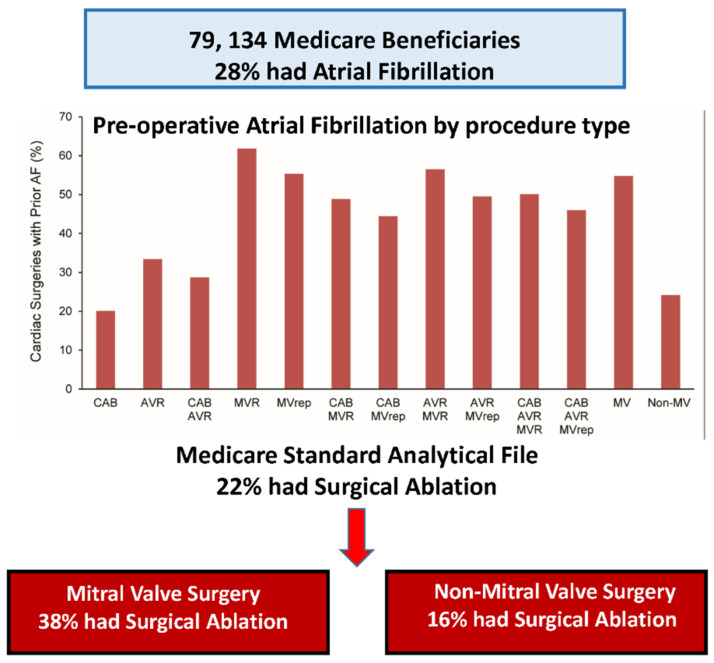
Of 79,134 Medicare patients undergoing surgery for various cardiac conditions that required surgery, 28% had associated atrial fibrillation. Only 38% of the patients undergoing mitral valve surgery and only 16% of patients undergoing non-mitral valve surgery had concomitant surgical ablation of their atrial fibrillation (reproduced with permission from McCarthy) [48].

Our group recently published an analysis of the prevalence of AF in patients undergoing cardiac surgery and the frequency of concomitant AF ablation in those patients (Figure 16) [48]. In 79,134 Medicare patients undergoing cardiac surgery, 28% had atrial fibrillation but only 22% of that 28% had concomitant AF ablation. A total of 33% of patients with AF undergoing MVR/r had concomitant AF ablation but only 16% of non-mitral valve surgical patients with AF had a concomitant ablation procedure. Women were less likely to have concomitant AF and less likely to have surgical ablation when they did.

The fact that less than one-quarter of all patients with concomitant AF currently receive AF ablation led to a subsequent study by the Cardiothoracic Surgical Trials Network directed at identifying the barriers to treating AF during mitral valve surgery and inferentially, to its treatment in other types of cardiac surgery. Adult cardiac surgeons across two statewide collaboratives (Virginia and Michigan) were surveyed on their knowledge and practice regarding AF ablation [49]. 80.3% of the respondents reported being “very comfortable” treating concomitant AF while 12.1% were not aware that the STS guidelines, the AATS expert consensus, and the Heart Rhythm Society expert consensus all rate the recommendation of treating concomitant AF as Class 1, Level A [50,51,52]. The most commonly cited barriers to treating concomitant AF were the following:

1. No barriers (40.3%);
2. Adds additional aortic cross-clamp time (22.7%);
3. “My patients are too high risk” (12.9%);
4. No additional payment for AF ablation (4.6%);
5. Not comfortable with AF ablation (3.0%);
6. Inadequate equipment (3.0%);
7. Inadequate staff and support (3.0%);
8. Belief that AF ablation worsens AF (3.0%);
9. Belief that AF ablation does not work (1.5%).

### 4.2. Safety of Concomitant AF Ablation in Cardiac Surgery Patients 

The above barriers are difficult to understand in view of the multiple publications in the surgical literature that clearly answer all of those concerns. Barriers 2 and 3 address the concern that adding concomitant AF ablation to other cardiac surgical procedures will increase the operative mortality. However, in 2012, Ad et al. showed that adding a full maze procedure did not affect the operative morbidity or mortality of CABG or AVR procedures [53] and in 2017 Al-Atassi et al. [54] showed that concomitant AF ablation did not increase the surgical risk in patients undergoing isolated CABG, AVR, or combined CABG and AVR. In 2013, Damiano’s group showed that adding a maze-IV procedure in patients undergoing mitral valve surgery did not affect operative mortality [55]. Badhwar et al. subsequently reported on 86,941 patients in the STS Adult Cardiac Surgery Database and showed that concomitant AF ablation actually reduced the operative mortality for mitral valve surgery while not impacting operative morbidity [5]. These findings are all recognized in the STS guidelines and AATS expert consensus statement [50,51]. Thus, it is clear that surgeons’ concerns about the risk of adding a concomitant AF ablation procedure to CABG, MVR/r, AVR/r, or combined CABG and AVR are unwarranted.

### 4.3. Efficacy of Concomitant AF Ablation in Cardiac Surgery Patients

Barriers 8 and 9 above indicate that nearly 5% of surgeons believe that concomitant AF ablation either does not work or actually makes the AF worse. However, Louagie et al. reported in 2009 that concomitant AF ablation restores sinus rhythm in over 90% of patients [56]. Furthermore, there have been seven prospective randomized controlled trials evaluating the efficacy of AF ablation and all seven showed significantly higher levels of postoperative sinus rhythm in patients treated with a variety of energy sources compared to untreated patients (Table 2) [27,57,58,59,60,61,62,63].

### 4.4. Beneficial Effects of Concomitant AF Ablation in Cardiac Surgery Patients

The restoration of sinus rhythm postoperatively has many positive benefits over leaving patients in AF or performing an AF ablation procedure that is unsuccessful. 

**Decrease in long-term strokes:** in 2003, Damiano’s group reported the 15-year follow-up of patients who underwent the cut-and-sew maze-I, maze-II, or maze-III procedures by the senior author (JLC) [64]. Over that 15-year period, the freedom from long-term stroke was 99.3%. Only one patient had a thromboembolic stroke and it was diagnosed as an incidental finding on a routine brain CT scan performed several years after the initial operation. In 2006, Itoh et al. reported a marked decrease in long-term stroke at 12 years following surgery in which the AF was ablated [65]. In 2019, Ad et al., showed that the long-term freedom from stroke following AF ablation was independent of whether or not mitral valve surgery was also performed [66]. Moreover, in 2019, Malaisrie et al., from our group reported on the 6-year follow-up of 24,069 patients in the STS database undergoing CABG [67]. Concomitant ablation of AF showed a significant decrease in long-term strokes compared to CABG patients with AF in whom the AF was ignored. The STS guidelines [50] and the AATS expert consensus statement [51] document the decrease in both perioperative strokes (Class IIa, Level A) and long-term strokes (Class IIa, Levels A, B-NR).

It has long been recognized that the left atrial appendage (LAA) is frequently the source of stroke in patients with AF, and perhaps as high as 90% of these strokes originate in the LAA [68,69]. Furthermore, these strokes tend to be more disabling or fatal compared to other sources of strokes (AHA). Therefore, surgeons, for many years, have treated the appendage by excising it, suturing it closed (either internally or externally), stapling it closed, or externally occluding it with other devices [68,69,70,71]. However, the success of these techniques has sometimes fallen short with suture closures reopening over time in as many as 60% [72], and staplers, sutures, or excision leaving an internal LAA “stump” over 10 mm which could potentially serve as another [69,72]. The most successful current approach appears to be closure with a clip device [71]. In concomitant surgery we place the clip while the heart is empty, on cardiopulmonary bypass, which allows the surgeon to place the clip very low (but avoiding the circumflex artery) and a residual stump over 10 mm can be avoided in almost every patient that way. In addition, the clip leads to gradual necrosis of the LAA, which becomes electrically silent and will not generate or propagate AF [73]. Studies on the LAA as a potential target for catheter ablation in patients with AF are ongoing (ASTRO AF, NCT04056390), and prior studies suggest this may be a benefit [74]. The LAAOS III Trial randomized 4770 patients with AF undergoing concomitant surgery to LAA closure versus no closure. There was a significant reduction in strokes at a mean follow-up of 3.8 years (7.0% vs 4.8%, hazard ratio 0.67). For patients with AF undergoing cardiac surgery it is clear that adding LAA closure is a worthwhile endeavor with no increase in peri-operative risk. The importance of catheter ablation of the LAA is currently undergoing further investigation.

**Improved long-term survival:** in 2012, our Northwestern University group reported that successful ablation of concomitant AF in patients undergoing mitral valve surgery improved the 5-year survival rate compared to untreated AF [75]. Furthermore, the 5-year survival rate in patients whose AF was ablated was virtually identical to that in patients who had no AF prior to surgery. In 2018, Damiano’s group showed that AF ablation using the maze-IV procedure improved the 10-year survival rate compared to patients in whom the AF was ignored [76]. In 2019, two important studies on survival improvement following concomitant AF surgery were published. Suwalski and the KROK investigators reported on the results of the Polish National Registry of Cardiac Surgery Procedures, which encompassed 11,381 patients with AF undergoing MV surgery between 2006 and 2017 in 37 centers across Poland [77]. After rigorous propensity matching between concomitant surgical AF ablation and no AF ablation, surgical ablation was associated with a nearly 20% improvement in long-term survival. In addition, the Northern New England Cardiovascular Disease Study Group reported the results of concomitant AF ablation from seven different centers. Concomitant AF ablation significantly improved the overall 5-year survival rates and it improved survival rates in three separate groups of patients, those undergoing isolated CABG, Valve Surgery, and combined CABG/Valve Surgery [78]. These three studies by Damiano’s group, Suwalski’s Polish Registry, and Iribarne’s New England consortium were all published after the 2017 STS guidelines and the 2017 AATS expert consensus statement, so the lack of current guideline recommendations based on improved survival following concomitant AF surgery is likely to change in the next version of the guidelines.

### 4.5. Comfort Level with AF Ablation Surgery

It is clear from the study mentioned above that one of the barriers to surgeons performing concomitant AF surgery is the fact that many are “uncomfortable” with performing any AF ablation procedure. This emphasizes a need for better surgical training and improved surgical education. The AATS formalized the first acknowledgement of this problem in their 2017 expert consensus statement that stated: “We highly recommend surgeons that are new to surgical AF be proctored by an experienced surgeon for three to five cases prior to performing surgical ablation alone (Class I, Level C)” [51]. They further recommended that “Optimization of patient outcomes requires a combination of education and formal training that incorporates understanding of the risks of leaving AF untreated, the risks associated with surgical ablation, the recommended procedure, including choice of lesion set and ablation technologies, and the results of surgical ablation.” Hopefully, with such encouragement, the percentage of patients treated for concomitant AF will increase in the future.

## 5. Current Status of Surgical Ablation for Stand-Alone AF

Because surgical intervention was introduced clinically some 11 years prior to the 1998 Haissaguerre article [12], stand-alone AF was initially treated solely by the maze procedure because it was the only interventional procedure available between 1987 and 1998. In fact, the first 100 or so maze procedures were performed solely for stand-alone AF because we were concerned that adding a maze procedure to CABG, MVR/r, or AVR surgery might increase the operative risk due to prolonged cardiopulmonary bypass time and aortic cross-clamp time. In the early 1990s, both the first co-authors (PMM) and (JLC) began combining the maze procedure with MVR/r surgery, as well as with other cardiac operations, and we confirmed separately that adding the maze procedure did not adversely affect perioperative morbidity or mortality. Since the publication of Haissaguerre’s 1998 article, however, virtually all stand-alone AF has been treated interventionally by electrophysiologists, not by surgeons.

### 5.1. Minimally Invasive CryoMaze-III Procedure 

During the ensuing two decades, a few experienced arrhythmia surgeons have continued to perform occasional surgical procedures for stand-alone AF, especially for catheter ablation failures. Several new, less invasive AF surgical procedures were introduced in an effort to make surgery more attractive for stand-alone AF treatment, including the minimally invasive CryoMaze-III procedure that we began using routinely in 1997 and first reported in 2000 [18]. The lesions in the minimally invasive CryoMaze-III procedure were identical to those of the previous cut-and-sew maze-III procedure performed through a median sternotomy and the results were the same. While the morbidity associated with the minimally invasive CryoMaze-III was less than that for the median sternotomy cut-and sew maze-III procedure, it still required the use of cardiopulmonary bypass, which remains the primary deterrent to its widespread use today. Nevertheless, using essentially the same minimally invasive maze-III procedure for the past 20+ years, Ad has attained 73% sinus rhythm off antiarrhythmic medication and 81% off anticoagulation at 5-years, exemplary results for the interventional treatment of stand-alone, long-standing persistent AF [79]. The results of surgery for stand-alone Cox maze procedures have continued to be superior to catheter ablation, but resistance to the expansion of surgery for stand-alone AF has persisted due to the invasiveness of surgery compared to that of catheter ablation.

Recently, the first author (PMM) developed a new technique to perform the Cryo-Maze-III procedure when employed concomitantly with other cardiac surgery [80]. In this new modification, a long flexible cryoprobe is pre-formed so that multiple lesions of the maze-III pattern can be accomplished. This results in the need to apply only three separate cryolesions in the left atrium and three in the right atrium, greatly reducing the time required to complete all of the lesions of the cryosurgical maze-III procedure.

### 5.2. Off-Pump Minimally Invasive Procedures

**Wolf mini-maze procedure:** this procedure was first reported in 2005 as an “off-pump” surgical treatment for AF [81]. Its name suggested that it was related to the maze procedure; it was simply a PVI plus staple-removal/occlusion of the left atrial appendage (LAA) and had nothing to do with the concept of using a maze pattern of lesions to treat AF. It proved to have results similar to those of catheter ablation for PAF but like catheter PVI, it was also suboptimal for the treatment of persistent and long-standing persistent AF. Thousands of patients underwent this surgical procedure, which required opening both chests and performing two separate pericardiotomies for AF with results similar to those that can be attained by much less invasive RF catheter ablation. 

**Dallas lesion set:** similarly, the Dallas lesion set, first reported in 2009 [82], was another “off-pump” surgical procedure for AF in which no right atrial lesions were performed and the combined mitral line/coronary sinus lesions of the maze-III were replaced with a single anterior lesion across Bachmann’s Bundle. Since the purpose of the mitral line/coronary sinus lesions is to preclude postoperative peri-mitral atrial flutter, this seemed to be a good idea. However, there were several problems with the Dallas Lesion Set. First, it divided Bachmann’s Bundle, leading to the possibility of delaying atrial conduction from the RA to the LA much as we had seen earlier with the original maze-I procedure. If inter-atrial conduction is delayed by as much as 150 msec, the LA and LV could be activated at the same time, resulting in the LA contracting against a closed mitral valve and eliminating any contribution of the LA to AV synchrony and forward cardiac output. This problem led to moving the anterior lesion more laterally with its proximal end at the base of the LAA and its distal end at the left fibrous trigone. The placement of the anterior lesion more laterally in the LA solved the problem of delayed inter-atrial conduction but it introduced another problem of having to perform extensive RF ablation in the region of the left fibrous trigone in immediate proximity to the left main coronary artery. In addition, the anterior lesion, which had to be performed epicardially with unipolar RF in the beating, working heart often failed, in which case the incomplete lesion often became arrhythmogenic. Finally, the elimination of all RA lesions in the Dallas Lesion Set was doomed to fail at least 30% of the time for persistent and long-standing persistent AF because 30% of the drivers that sustain AF are located in the RA. 

The Wolf mini-maze procedure and the Dallas lesion set were the two most widely employed of the numerous minimally invasive surgical approaches to the treatment of AF developed in the past 20 years. Virtually every lesion pattern one could think of was at one time or another used to treat AF, none of which had a sound scientific basis based on rigorous mechanistic studies. As a result of the poor results obtained for catheter ablation and the disappointment with the new minimally invasive surgical procedures, a new concept evolved of performing so-called “hybrid” procedures for long-standing persistent AF in which interventional electrophysiologists and surgeons work together to overcome the problem of long-standing persistent AF without requiring cardiopulmonary bypass.

### 5.3. Off-Pump Hybrid Procedures 

Four general approaches have been popularized as hybrid procedures over the past decade, each of which can be performed as either a “joint” procedure or a “staged” procedure. They include:

(1) Muneretto/Bisleri thoracoscopic (TT) maze procedure;
(2) van Putte TT modified maze procedure;
(3) LaMeir TT maze procedure based on intraoperative mapping;
(4) Convergent procedure.

**Figure 17:**
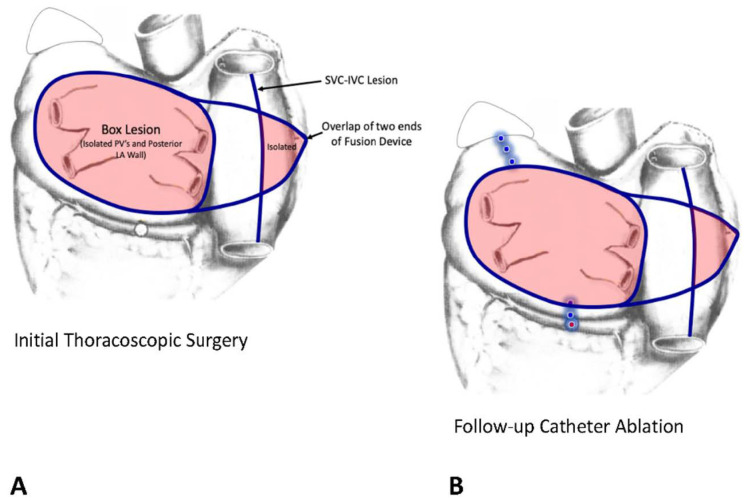
The lesion patterns of the Muneretto/Bisleri hybrid procedure. (**A**) During the initial thoracoscopic surgical procedure, a box lesion encircling all four pulmonary veins and the posterior wall of the left atrium is created with the fusion device to isolate that portion of the left atrium. The long arms of the Fusion device are extended across to the right atrium and overlapped. The SVC-IVC lesion then results in isolation of a portion of the right atrial free wall. (**B**) At the time of the follow-up catheter ablation, the surgical lesions are “touched-up” as needed and additional catheter lesions are created as needed. This diagram shows catheter lesions (round dots) across the Coumadin Ridge between the left superior pulmonary vein and the orifice of the left atrial appendage and the mitral line and coronary sinus lesions across the left atrial isthmus between the lower pulmonary veins and the posterior mitral valve annulus.

**Muneretto/Bisleri hybrid TT procedure:** this procedure is performed through three small stab wounds in the right chest, one for the thoracoscope and two for the surgical instruments. The PV’s are encircled with the epicardial Fusion device (AtriCure Inc., 7555 Innovation Way, Mason, OH 45040 USA) that creates a box lesion around all four PV’s and the posterior LA wall. The alleged advantage of the Fusion device is that it can create a vacuum that “sucks” the atrial wall up into the device so that both bipolar and unipolar RF can then be used to create the lesion. Muneretto also extends the tips of the Fusion device over to the free-wall of the right atrium where he crosses them to overlap one another. He then creates an RF lesion from the SVC to the IVC that results in the isolation of a significant portion of the RA free-wall. 6 weeks later, the EP performs a follow-up EP study in which he “touches up” any gaps in the epicardial surgical lesions and adds any additional lines that might be required based on the interval history of arrhythmias and on intraoperative mapping (Figure 17). In the prospective, randomized HISTORIC-AF trial, the 1-year success rate for this hybrid procedure was 88% in patients with stand-alone, persistent, and long-standing persistent AF [83].

**Figure 18:**
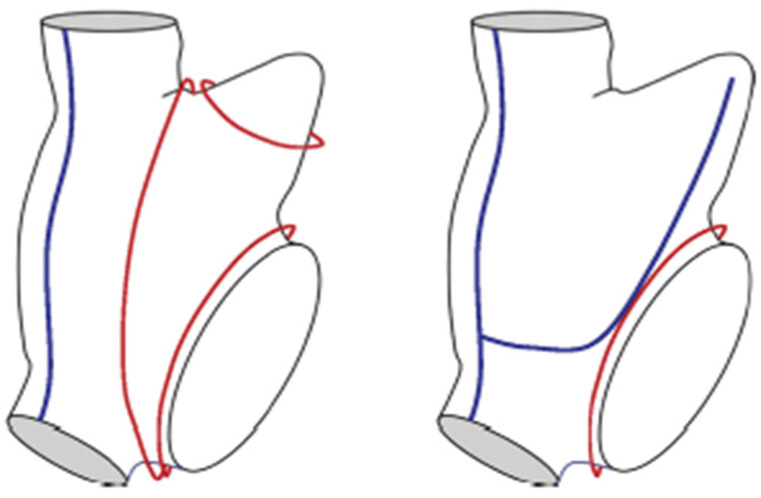
The blue lines represent the right atrial lesions in the van Putte totally thoracoscopic (TT) procedure. The red lines represent potential macro-reentrant drivers of atrial fibrillation that can occur in the right atrium. Note that the two lesions that can be created thoracoscopically do not preclude the development of a macro-reentrant driver around the annulus of the tricuspid valve. However, this can be accomplished by having the EP create a cavotricuspid isthmus (CTI) lesion at the time of the follow-up catheter ablation procedure.

**The van Putte hybrid TT procedure:** in this approach, the surgeon performs bilateral thoracoscopic procedures with three stab wounds in each chest [84]. The left thorax is entered first and the PV’s are isolated with a bipolar RF clamp. Next, partial roof and floor lesions are created from the right side with the dual-electrode unipolar Coolrail linear pen. In recent procedures, van Putte has added two lesions in the RA, one between the SVC and IVC and another from this intercaval line across the lower RA and then curving it upwards to the tip of the RA appendage as recommended in the hybrid maze procedure (Figure 18) [84]. The patient is then turned over and the thoracoscope is inserted into the left chest. Through this exposure, the left PV’s are isolated with a bipolar RF clamp and the roof and floor lesions are completed with the Coolrail pen (AtriCure, Inc., 7555 Innovation Way Mason, OH 45040 USA). An anterior LA lesion to preclude peri-mitral atrial flutter (A.K.A. “atypical left atrial flutter”) is then extended from the roof lesion to the left fibrous trigone as far to the left as possible to avoid transection of Bachmann’s Bundle. Because transmurality is so difficult to attain with this lesion, it is often incomplete and becomes arrhythmogenic. As a result, van Putte has eliminated this lesion from his technique. Finally, the LA appendage is occluded with an epicardial AtriClip.

van Putte performs this procedure as a definitive treatment, not as a true hybrid procedure, so the follow-up catheter ablation is not mandatory. At Northwestern University, we insist that the patient undergo this follow-up EP study with any required additional catheter “touch up” and/or ablation. Our experience with this TT modified maze procedure in the first 20 patients (67% long-standing persistent AF) has been excellent. At one-year, the freedom from atrial flutter/fibrillation was 95%. The one patient with recurrent atrial fibrillation refused to submit to the follow-up catheter study and potential ablation. Moreover, van Putte’s group recently reported an overall freedom from atrial arrhythmias in 82 patients undergoing only the thoracoscopic procedure of 60% after a mean follow-up of 4.0 + 0.3 years [85].

**Figure 19:**
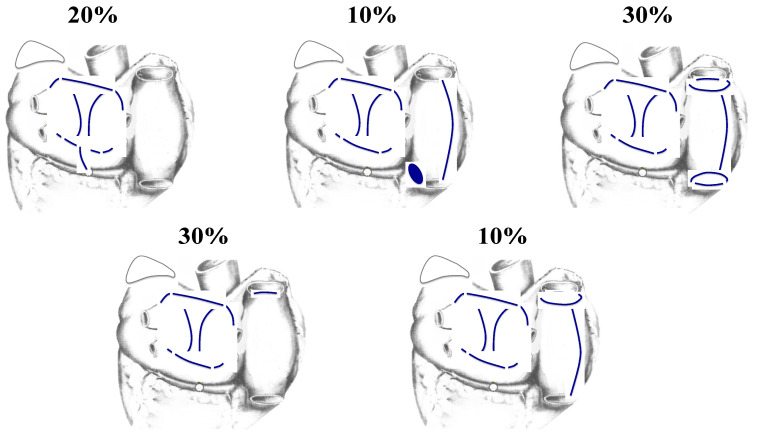
Because the La Meir hybrid approach depends on intraoperative mapping, different patients receive different patterns of lesions during the surgical phase of the hybrid procedure. These are the lesion patterns and their frequency reported by La Meir et al. in their first publication on their hybrid technique [86]. Many authorities are skeptical of the long-term success of map-guided techniques for the treatment of long-standing persistent atrial fibrillation because the pattern of atrial activation can vary from one day to the next.

**LaMeir TT hybrid procedure based on intraoperative mapping:** the question of whether or not any interventional procedure can be based on intraoperative mapping has persisted since the development of surgery for AF in the mid-1980s when we learned that AF surgery could not be map-guided. The most utilized hybrid procedure that counters that observation has been the approach popularized by Mark LaMeir and associates in Brussels and Maastricht [86], De Groot’s Amsterdam group also uses intraprocedural mapping, but is somewhat less dependent on it than on the anatomy of the atrium in that they routinely perform PV antral isolation and ganglionic plexus ablation in all procedures [87]. In LaMeir’s approach, the lesion pattern for each patient is based on the intraoperative mapping findings in that specific patient and therefore, the lesion patters vary from one patient to another (Figure 19) [86]. LaMeir reported that in 72 patients with persistent AF, 75% were free of atrial arrhythmias at 1-year. DeGroot reported in a Letter-to-the-Editor that 88% of their 66 patients were in sinus rhythm at 5 years, though only 55% of them had not experienced a recurrence of AF during the 5-year follow-up. Recently, LaMeir has reported a hybrid technique in which he performs the entire TT procedure through the left chest [88]. He reports a success rate of 68.8% at a mean follow-up of 24.9 + 11.8 months in 51 patients.

**Figure 20:**
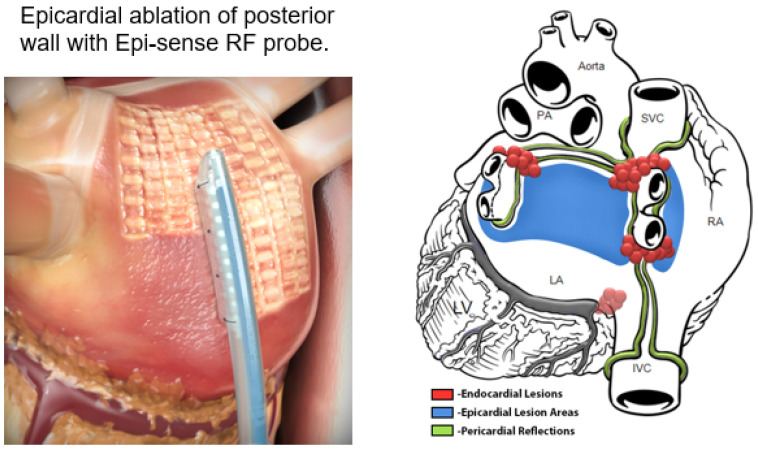
The convergent procedure: left panel: epicardial ablation of the posterior left atrial (LA) wall using the Epi-Sense radiofrequency (RF) ablation device (AtriCure Inc., 7555 Innovation Way, Mason, OH 45040, USA). Right panel: the green lines show the epicardial reflections that limit where the epicardial RF can be accomplished. The blue area represents the multiple sites of epicardial RF ablation. The red dots represent the sites of endocardial catheter ablation that completes the pulmonary vein isolation and the cavotricuspid isthmus (CTI) lesion in the right atrium (used with permission from AtriCure, Inc.).

**Convergent procedure:** the “ex-maze procedure” was the first technique to be introduced and while it is no longer performed, it evolved into what is now known as the “Convergent Procedure”, which has become quite popular with both surgeons and EP’s. The original ex-maze procedure was performed through an abdominal–transdiaphragm approach [89], while the current convergent procedure is performed via a subxiphoid approach [90]. Using the Epi-Sense RF probe (AtriCure Inc., 7555 Innovation Way, Mason, OH 45040 USA) epicardially, the surgeon first ablates the posterior wall of the left atrium epicardially (Figure 20) through a small subxiphoid incision and pericardiotomy. Thoracoscopic occlusion of the left atrial appendage with an epicardial AtriClip (AtriCure Inc., 7555 Innovation Way, Mason, OH 45040 USA) may or may not be included. Either jointly or a few weeks later, the EP then performs an endocardial PVI with or without a CTI lesion in the right atrium.

Recently, a prospective randomized controlled trial was reported in which the hybrid Convergent Procedure was compared to catheter ablation only for the treatment of persistent and long-standing persistent AF [91]. The hybrid Convergent Procedure showed superior effectiveness compared to endocardial catheter ablation with freedom from atrial arrhythmias absent new or increased dosage of previously failed class I/III antiarrhythmic drugs of 67.7% versus 50.0%, respectively (risk ratio, 1.35, *p* = 0.036), and off antiarrhythmic drugs success of 53.5% versus 32.0%, respectively (risk ratio, 1.67, *p* = 0.0128). At 18 months using 7-day Holter monitoring, 74% of the subjects receiving the hybrid Convergent Procedure had at least a 90% AF burden reduction compared to 55% with endocardial catheter ablation only (risk ratio, 1.34, *p* = 0.0395). This study has led to a resurgence in the utilization of the hybrid Convergent Procedure to treat long-standing persistent AF.

**Figure 21:**
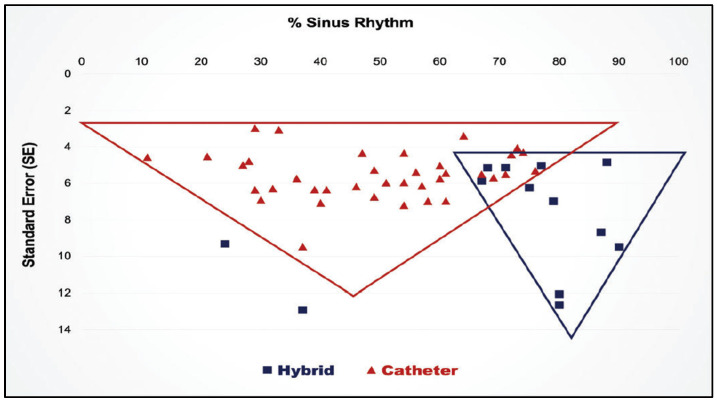
Results of a meta-analysis the superiority of hybrid procedures over catheter ablation for long-standing persistent atrial fibrillation. (Reproduced by permission from van der Heiiden) [83].

Finally, in 2019 a systematic review and meta-analysis comparing hybrid procedures with catheter ablation for long-standing persistent AF showed a clear superiority for the hybrid procedures (Figure 21) [92].

## 6. Future of the Interventional Treatment of Atrial Fibrillation

### 6.1. Future of Catheter Ablation for Atrial Fibrillation

Recently two new and promising energy sources have been introduced for the catheter ablation of AF, pulsed field ablation [93] and ultra-low cryothermia [94].

**Pulsed field ablation (PFA):** this approach takes advantage of an energy source that can be delivered to tissues in both a reversible and irreversible form. Reversible electroporation has been used to treat cancer by increasing the size of the natural openings in cell walls to allow small molecules of a desired compound (e.g., anti-cancer drugs) to be inserted into the cells. It is a non-heat-based energy source that can be simply turned off after the insertion of the desired drug into the cells, allowing their natural cell wall openings to return to their normal size with no permanent injury to the cells themselves. Irreversible electroporation can be accomplished by increasing the strength of the energy applied, which destroys the cell walls and therefore, is capable of creating permanent lesions in the target tissue. When used to create lesions in the atrial myocardium its major advantage is that it appears to be “tissue-specific”, thus avoiding collateral damage to adjacent tissues such as nerve or esophageal tissue. The safety of irreversible electroporation (pulsed field ablation, or PFA) is the most attractive feature of this new energy source for creating atrial lesions to treat atrial fibrillation.

There are some problems, however, with PFA. First, it is often difficult to achieve penetration of the atrial wall from the endocardium to greater depths than 3–4 mm. This may be due to “scatter” of the energy as it travels through fibrotic atrial muscle or it may be due to the loss of much of the PFA energy to the intracavitary blood. Since blood has a lower resistance than atrial muscle, most of the PFA energy applied to the atrial endocardium is lost into the blood stream as evidenced by the numerous “bubbles” that are easily seen on echo during PFA application endocardially. Nevertheless, PFA has created the greatest excitement of any new energy source for interventional AF therapy since catheter ablation was introduced for the treatment of atrial fibrillation in 1998.

**Ultra-low cryoablation (ULC):** the only cryosurgical devices available since they were introduced into cardiac surgery in the mid-1970s have utilized internally expanding nitrous oxide as the cryogen, which cooled the cryoprobe to approximately −600 C. Some 30 years later, argon gas, capable of cooling the cryoprobe to approximately −1600 C, was introduced as a cryogen that could be used in surgical instruments but not for catheter ablation. Even today, available cryocatheters and cryoballoons still use nitrous oxide as the cryogen, though surgeons can choose between commercially available nitrous oxide and argon surgical cryoprobes.

It has long been known that the coldest cryogen in nature is liquid nitrogen, which is capable of reaching a temperature of −1960 C. Unfortunately, liquid nitrogen delivery requires such large probes and tubing for delivery that it is not feasible as a clinical tool for catheter ablation. However, several years ago it became possible to suspend liquid nitrogen in a so-called “near-critical” phase between liquid and gas. This provided a cryogen with the viscosity of gas that can be delivered through extremely small conduits at a temperature of −1960 C, making it feasible as a cryogen for use in ablation catheters. For the past 11 years, Adagio Medical, Inc. of Laguna Hills, CA has been developing a cryocatheter system that provides not only the lowest possible temperature of −1960 C, but also a catheter that can create a continuous linear cryolesion in the atrium. Because of the extremely low temperature, the lesions are uniformly transmural and contiguous with no gaps throughout the linear lesions. This provides the EP’s with a revolutionary tool for creating reliable lesions of conduction block anywhere in either atrium.

After years of pre-clinical studies, two initial clinical trials using this system have now been completed. The first was directed at creating CTI lesions for classic atrial flutter and the second clinical trial was designed to treat persistent and long-standing persistent AF. The time required to perform complete isolation of the PVs, including the posterior LA wall, the mitral line, and the CTI lesion is measured in minutes. The results thus far have been spectacular but await publication in a peer-reviewed journal.

**Combined PFA and ULC (PFCA):** finally, the same small company in California has developed a system by which both PFA and ULC can be delivered through the same ablation catheter to take advantage of the positive characteristics of both energy sources. Once the ablation catheter is positioned in the desired location against the atrial endocardium, low-level cryothermia is initiated to “fix” the catheter in position and to form an insulator around the catheter itself. PFA can then be delivered through the same catheter to take advantage of its “tissue specific” characteristic. The “ice-ball” insulator around the catheter prevents the loss of PFA energy into the atrial cavitary blood and allows all of the PFA energy to be directed solely into the atrial wall. Based on the early results of the pre-clinical and clinical trials, it is likely that PFCA will become the energy source combination of the future since it would appear to overcome the major limitations of the unreliable and incomplete lesions currently created by the tip of available ablation catheters. The pre-clinical and clinical studies using PFCA are being conducted primarily by Dr. Atul Verma. 

### 6.2. Future of Surgical Ablation for Atrial Fibrillation

The concomitant surgical ablation of atrial fibrillation in patients undergoing CABG, MVR/r, and AVR will likely continue to grow in the near and distant future. Studies over the past 10 years have documented that treating both the primary cardiac problem and any associated AF does not increase the operative risk and in fact, may actually decrease the operative mortality compared to ignoring the AF. While less than one-half of all such patients now receive concomitant AF ablation, the STS Guidelines and the AATS Expert Consensus both declared that concomitant AF ablation is a Class I recommendation, and Class IIa in the valve guidelines. Therefore, the reluctance of some surgeons and their lack of understanding of when and how to perform concomitant AF ablation procedures can be overcome by continued improvement in their education and understanding of the AF problem. The advantages of adding concomitant AF ablation to CABG, MVR/r, and AVR procedures have been documented in multiple publications and can no longer be ignored by surgeons if they wish to provide their patients with the standard of care that they deserve. At a minimum, following the LAAOS III randomized trial, secure closure of the LAA should become a Class I recommendation and incorporated in every concomitant operation when practical. Moreover, the magnitude of the problem of AF worldwide suggests that in the future, concomitant AF ablation will likely become a major part of every cardiac surgeon’s clinical practice. It is our hope that using the more efficient, and very effective, cryoablation lesion set that we pioneered at Northwestern will address the increased cross clamp and cardiopulmonary bypass times of the maze-IV procedure. This simpler approach should also increase adoption, especially if wider FDA approval for labelling (ICE-AF ClinicalTrials.gov Identifier: NCT03732794) is approved.

The future of surgery for stand-alone AF is also promising. Despite the development of newer and more effective ablation catheters, the problem of chronic AF present for many months or years in patients with large left atria will only increase in the future as AF becomes more prevalent. Therefore, the future for relatively simple and non-invasive surgical/catheter hybrid approaches such as the Convergent Procedure looks bright, as there is little doubt that there will always be a need for some degree of surgical intervention, as well as catheter ablation, for stand-alone long-standing persistent AF. However, it is likely that as these new technologies and techniques are perfected, fewer patients will undergo the maze-III/IV procedure that requires cardiopulmonary bypass. Perhaps the most advantageous development that could occur in our future struggle against the worsening AF epidemic will be the development of functional heart teams consisting of EP’s, arrhythmia surgeons, nurse specialists, and other ancillary personnel who are devoted to the diagnosis, treatment, and long-term follow-up of patients with atrial fibrillation.

## Figures and Tables

**Table 1:** Rates of specific complications by indication for catheter ablation from 2000 to 2013 (reproduced with permission from Hosseini) [6].

	AF	AFL	VT	SVT
Catheter ablation procedures (unweighted)	39,562	25,723	9642	33,346
Catheter ablation procedures (weighted)	190,398	123,163	46,495	159,895
≥1 complication	7.21	3.91	9.90	3.29
Mortality	0.24	0.20	1.82	0.12
Post-procedural stroke or TIA	0.31	0.19	0.38	0.14
Post-procedural infection	0.26	0.32	1.07	0.18
Cardiac complications ^a^	1.15	0.53	1.24	0.68
Pericardial complications	2.02	0.41	2.47	0.81
Vascular complications	1.09	0.64	2.08	0.48
Hemorrhage	3.64	2.04	3.67	1.37
Hemorrhage requiring transfusion	0.69	0.37	0.86	0.18
Diaphragm paralysis	0.11	0.05	0.02	0.04
Pneumothorax or hemothorax	0.14	0.09	0.04	0.15
Length of stay	2.63 ± 0.06	3.18 ± 0.05	4.7 ± 0.09	2.22 ± 0.04

Values are % or mean ± SEM. *p* < 0.05 considered significant. ^a^ Includes post-operative cardiac block, myocardial infarction, cardiac arrest, and congestive heart failure. AF = atrial fibrillation; AFL = atrial flutter; SVT = supraventricular tachycardia; TIA = transient ischemic attack; VT = ventricular tachycardia.

**Table 2:** Seven prospective randomized trials showing the efficacy of concomitant AF surgery in converting patients to normal sinus rhythm postoperatively.

Trial, Year	Number of Pts	Technology	Control 12 Month NSR	Treated 12 Month NSR
Deneke et al., 2002	30	Unipolar Cooled RF	26.7%	80% (*p* < 0.01)
Schuetz et al., 2003	43	Microwave	33.3%	80% (*p* = 0.036)
Akpinar et al., 2003	67	Unipolar RF	9.4%	93.6% (*p* = 0.0001)
Abreu Filho et al., 2005	70	Unipolar Cooled RF	26.9%	79.4% (*p* = 0.001)
Doukas et al., 2005	101	Unipolar RF	4.5%	44.4% (*p* = 0.001)
Blomström-Lunqvist 2007	69	Cryoablation	42.9%	73.3% (*p* = 0.013)
Chevalier 2009	43	Unipolar RF	4%	57% (*p* = 0.004)
Gillinov, 2015	260	Cryoablation	29%	63.2% (*p* < 0.001)
